# Impact of Photodynamic Diagnosis on Immune Cell Infiltration in BCG-treated Non–muscle-invasive Bladder Cancer

**DOI:** 10.1016/j.euros.2026.07.006

**Published:** 2026-08-08

**Authors:** Tine Ginnerup Andreasen, Trine Strandgaard, Tessa Jane Divita, Kristine Young-Halvorsen, Anders Neijber, Lars Dyrskjøt

**Affiliations:** a Department of Molecular Medicine, Aarhus University Hospital, Aarhus, Denmark; b Department of Clinical Medicine, Aarhus University, Aarhus, Denmark; c Photocure ASA, Global Medical Affairs and Clinical Development, Oslo, Norway

**Keywords:** Bladder cancer, Bacillus Calmette-Guérin, Immune tumor microenvironment, Photodynamic diagnosis

## Abstract

**Background and objective:**

Photodynamic diagnosis (PDD) is used during transurethral resection of bladder tumors (TURBT) to improve detection of non–muscle-invasive bladder cancer (NMIBC). Emerging evidence suggests that PDD may also influence immune activation. We investigated whether PDD is associated with immune-related changes in tumors and urine from patients with NMIBC receiving Bacillus Calmette-Guérin (BCG) immunotherapy.

**Methods:**

We retrospectively analyzed paired pre- and post-BCG samples from 156 patients with NMIBC with detailed clinical data. PDD exposure was determined from medical records and defined as PDD use within 100 days before BCG induction. PDD status was linked to existing multi-omics data, including urine proteomics (Olink), tumor ribonucleic acid (RNA) sequencing, and imaging mass cytometry (IMC) for spatial immune profiling.

**Findings and limitations:**

Urine proteomics showed post-BCG upregulation of immune-related proteins, including programmed death-1 and vascular endothelial growth factor receptor-2, in PDD-exposed patients. In contrast, RNA sequencing suggested increased post-BCG infiltration of multiple immune cell populations (B cells, T cells, cytotoxic cells, and mast cells) in No PDD patients but not in PDD-exposed patients. Consistently, IMC indicated increased infiltration of cluster of differentiation (CD)4⁺, CD8⁺, and natural killer cells post-BCG in No PDD exposed patients, with minimal changes in PDD-exposed patients. The exploratory design and potential confounding limit causal interpretation, and no clear association with clinical outcomes was demonstrated.

**Conclusions and clinical implications:**

This exploratory and biologically focused study suggests that while PDD appears to modulate the local urinary proteome, no clear shifts in tumor immune cell infiltration were observed. These findings do not support a consistent effect of PDD on the tumor immune microenvironment, and no association with clinical outcomes was observed. Determining whether the added benefit of PDD stems purely from enhanced diagnostics or includes a direct immunomodulatory effect warrants further investigation.

## Take Home Message

We investigated biological changes associated with photodynamic diagnosis (PDD) in patients with non–muscle-invasive bladder cancer treated with Bacillus Calmette-Guérin (BCG). PDD exposure was associated with distinct post-BCG urinary immune proteomic changes, including increased programmed death-1 and vascular endothelial growth factor receptor-2.

ADVANCING PRACTICE **What does this study add?**

This study investigates the association between photodynamic diagnosis (PDD) prior to Bacillus Calmette-Guérin (BCG) immunotherapy and immune-related changes in non–muscle-invasive bladder cancer using a multi-omics approach. PDD exposure was associated with distinct post-BCG urinary immune proteomic changes, including increased levels of programmed death-1 and vascular endothelial growth factor receptor-2, without corresponding increases in intratumoral immune cell infiltration. In contrast, patients not exposed to PDD showed pronounced post-BCG immune cell infiltration in tumor tissue.

**Clinical Relevance**

Although photodynamic diagnosis may induce measurable immune-related changes in the urinary milieu, this study found no evidence that PDD enhances the intratumoral immune response to BCG or improves oncological outcomes. Clinicians should therefore continue to use PDD primarily as a tool to improve tumor detection and resection quality, while any potential immunomodulatory effects remain investigational. Associate Editor: M. Carmen Mir, MD; PhD

**Patient Summary**

We examined whether the use of a light-based imaging technique during bladder tumor removal is associated with differences in the immune response during subsequent BCG treatment. We observed changes in immune-related proteins in urine after BCG in patients who had undergone photodynamic diagnosis, while changes in immune cell infiltration within tumor tissue were more pronounced in patients who had not received this technique.

## 1 Introduction

Bladder cancer is a prevalent malignancy characterized by a high risk of recurrence, necessitating rigorous, and long-term surveillance. Approximately 75% of cases are diagnosed as non–muscle-invasive bladder cancer (NMIBC), for which diagnosis and initial treatment are typically performed concurrently via transurethral resection of bladder tumors (TURBT) [1], [2]. Cystoscopy remains the primary diagnostic modality for bladder cancer and allows for both visualization and endoscopic resection of visible lesions. To enhance tumor detection and ensure complete resection, advanced optical imaging techniques such as narrow band imaging and photodynamic diagnosis (PDD), also known as blue-light or fluorescence cystoscopy, have been developed to guide TURBT [2].

PDD involves intravesical instillation of a photosensitizing agent, such as hexaminolevulinate (HAL), which preferentially accumulates as photoactive porphyrins in malignant urothelial cells. Upon exposure to blue-violet light, these porphyrins fluoresce, enabling enhanced visualization of neoplastic lesions during cystoscopy. Guidelines for use of PDD includes (1) primary tumor to perform complete resection, (2) alternative to selected site biopsies to investigate for carcinoma in situ (CIS), (3) first control after Bacillus Calmette-Guérin (BCG) given based on CIS, and/or (4) positive urine cytology (Paris IV or V) with normal cystoscopy and CT [2]. Several studies have shown that PDD improves lesion detection, sensitivity, and recurrence-free survival in NMIBC compared to white-light cystoscopy. However, PDD has lower specificity and may yield false positives, particularly in the presence of inflammation, recent BCG, or chemotherapy instillations, or recent TURBT [2], [3], [4], [5], [6], [7], [8].

Adjuvant intravesical therapy with BCG is recommended following TURBT in patients with high-risk NMIBC, due to the substantial risk of disease recurrence and progression [2], [9]. Although TURBT aims to achieve complete tumor resection, residual microscopic disease, CIS, and field cancerization effects may contribute to recurrence. BCG treatment activates the host immune system, and therapy response is dependent on the landscape of the tumor microenvironment (TME), including the compositional and functional status of immune cells [10], [11]. Previous studies have suggested that PDD may be associated with immune activation, as observed in both animal [12] and human [13] models. These studies showed evidence of increased T-cell activity and reduced T-cell exhaustion. Lamy et al. demonstrated that HAL-mediated PDD, when paired with local immune checkpoint inhibition, augments immune stimulation through both local and systemic effects [12], [14]. This raises the possibility that combining PDD with another localized and immunologically active treatment such as BCG could potentially modulate immune cell infiltration within the tumor microenvironment and potentially enhance the therapeutic efficacy of BCG.

In this study, we performed an exploratory analysis to investigate whether PDD exposure is associated with differences in the local tumor microenvironment in patients with NMIBC treated with BCG. We analyzed urine and tumor samples to evaluate overall and cell-type specific immune cell infiltration. Specifically, we examined paired pre- and post-BCG samples from patients treated with either PDD-guided or white light–guided resection. Our analyses included Olink proteomics of urine samples, and transcriptomic profiling to assess immune-related gene expression of tumor tissue as well as imaging mass cytometry (IMC) spatial proteomics. These complementary approaches were used to explore the potential associations between PDD exposure and changes in the local immune landscape and their relevance to BCG response.

## 2 Materials and methods

### 2.1 Patient and sample collection

We retrospectively included 156 patients with NMIBC who received at least five out of six cycles of BCG. Tumor and urine samples were collected before and after treatment. Urine samples were collected up to 4 mo before or after BCG. Pre-BCG samples were collected a median of 43 days before treatment (interquartile range [IQR], 33–63), and post-BCG samples a median of 89 days after treatment (IQR, 75–105). Tissue samples covered a broader time frame, with pre-BCG specimens obtained near PDD/BCG exposure and most post-BCG samples collected years later (ribonucleic acid [RNA]: pre-BCG median 181 days [IQR, 58–543], post-BCG median 364 days [IQR, 204–929]; IMC/tissue microarrays (TMA): pre-BCG median 86 days [IQR, 55–233], post-BCG median 215 days [IQR, 98–360]). Given limited availability, they were included to maintain cohort size and representation (Supplementary Fig. 1). Post-BCG high-grade (HG) recurrence was defined as HG urothelial carcinoma recurrence within 2 yr after completion of BCG induction or progression to muscle-invasive bladder cancer at any time during follow-up. Follow-up was calculated from the time of pre-BCG RNA-sequenced (RNA-seq) tumor to end of follow-up, which was defined as the last of the following: last cystoscopy, last tumor detected, cystectomy, progression, or metastases.

### 2.2 Molecular data and analyses

Urine samples were analyzed using the Olink Target 96 Immuno-Oncology panel, yielding data from 169 samples (92 pre-BCG, 77 post-BCG, of these 60 paired samples). RNA-seq data were available from 149 tumors (108 pre-BCG, 41 post-BCG) as previously described (Strandgaard et al [15], 2022). TMA generated from paired tumor specimens were used for IMC analyses [16]. A regression-based T-cell exhaustion score was applied to assess associations between PDD use and T-cell exhaustion [15]. IMC was performed using the Hyperion platform (Standard Biotools) for 36 cancer- and immunology-related protein markers. Cell types were identified based on characteristic lineage marker expression (eg, cluster of differentiation [CD]14, CD16, PanCytokeratin, GATA3, FOXP3, CD4, CD8a, CD68). Iterative Leiden clustering was used to define distinct cell populations, which were manually annotated according to marker profiles. The resulting cell type abundances were extracted and used for downstream analyses.

### 2.3 PDD information

PDD status was determined for all patients by reviewing electronic medical records. Patients were classified as PDD exposed if they had received PDD during a cystoscopy or TURBT within 100 days prior to the initiation of BCG treatment. All pre-BCG samples selected for analysis were obtained before any PDD exposure to ensure they represented PDD-negative baselines. Patients who had received PDD more than 100 days before BCG initiation were excluded from the analysis to reduce potential confounding effects. Patients who had not received PDD at any time were classified as No PDD. A 100-d cutoff was used, reflecting both the data distribution, the maximum anticipated time window for any PDD-related influence and to ensure consistency across the cohort. The majority of patients who received PDD did so within this time frame (Supplementary Fig. 2). As PDD was not randomly assigned and is typically used in higher-risk disease, its use may be influenced by underlying tumor characteristics, including European Association of Urology (EAU) risk group and disease severity, which may also affect the local immune microenvironment. Differences in baseline tumor characteristics between groups were therefore anticipated and considered potential sources of confounding.

### 2.4 Statistical analysis

Statistical analyses were performed to address study questions related to urinary protein changes, transcriptional immune signatures, and spatial immune cell composition in relation to PDD status and BCG response. To evaluate urinary protein changes induced by BCG treatment in relation to PDD exposure, we analyzed paired pre- and post-BCG samples using Wilcoxon signed-rank tests. These analyses were performed using the olink_wilcox() function from the OlinkAnalyze R package, which accounts for within-patient dependence using patient identifiers. Multiple testing correction was applied using the Benjamini-Hochberg method to control the false discovery rate. In one supplementary analysis including only post-BCG samples, unpaired comparisons were performed using Wilcoxon rank-sum tests. Differences in RNA-based immune cell signatures, immune infiltration scores, T-cell exhaustion levels, and IMC-derived immune cell subsets were analyzed using Wilcoxon rank-sum tests for independent group comparisons. A multivariable Cox proportional hazards regression model was performed to assess associations with recurrence-free survival, adjusting for relevant covariates. The *p* values were reported as unadjusted, as these analyses were considered exploratory, and *p* values below 0.05 were considered statistically significant. R and RStudio was used for statistical analysis (R version 4.4.3 and RStudio version 2024.12.1+563).

## 3 Results

### 3.1 Patient characteristics

**Fig. 1:**
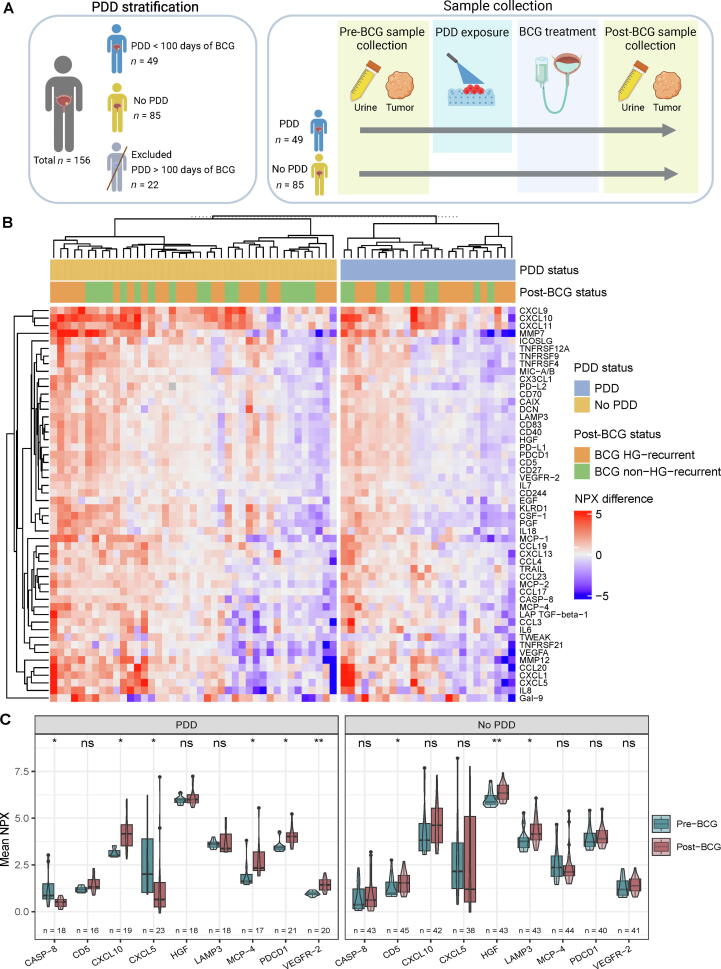
Study setup and urinary proteins. (A) Patients were stratified according to PDD exposure during diagnosis and treatment. Patients who had been exposed to PDD over 100 d before initiation of their BCG treatment were excluded (*n* = 22). Urine and tumor samples were retrospectively collected before and after BCG treatment. All included pre-BCG samples were PDD naïve. (B) Heatmap showing the difference in NPX values between paired pre- and post-BCG samples, stratified by PDD status. Columns represent individual patients; rows represent proteins. Red indicates a positive difference (increased NPX after BCG), while blue indicates a negative difference (decreased NPX after BCG). Hierarchical clustering of columns was based on Euclidean distance and complete linkage. (C) NPX of selected proteins. Proteins with significant pre- to post-treatment changes in patients after NPX values were filtered above the protein-specific median. Selected proteins from the Olink panel are shown, representing those with a significant change (Wilcoxon signed-rank test) between pre- and post-treatment samples in either the PDD or No-PDD group. The figure is split by clinical group (PDD vs No-PDD), with paired samples displayed for each protein. Significant differences are denoted with *, **, or *** if they are <0.05, <0.01 or <0.001, respectively. BCG = Bacillus Calmette-Guérin; NPX = normalized protein expression unit; PDD = photodynamic diagnosis. (A) is created with Biorender.com.

Of the included 156 patients, 49 had received PDD within 100 days of BCG (PDD group) and 85 had not received any PDD (No PDD group). A total of 22 patients were excluded because time from PDD to BCG exceeded 100 days (Fig. 1A). Post-BCG response was HG recurrent in 40% of patients within 2 yr (Table 1). When stratified by PDD status, we did not observe statistically significant differences in sex distribution, age at diagnosis, smoking status, follow-up time, or post-BCG response between the PDD and No PDD groups. However, there was a significant difference in EAU risk group distribution (*p* < 0.001), with the No PDD group having a higher proportion of intermediate-risk cases and the PDD group being enriched for high- and very high-risk tumors (Supplementary Table 1). This might reflect a higher risk profile, which is the reason PDD was elected for these patients. To account for potential confounding, a Cox proportional hazards regression analysis was performed. After adjustment for EAU risk group, age, and sex, no statistically significant association was observed between PDD status and BCG response (Supplementary Table 2, Supplementary Fig. 3). Table 1 lists the clinical characteristics of all the patients.

**Table 1:** Clinical characteristics of the patient cohort

Characteristic	*N* = 156^a^
**Sex (male)**	123 (79%)
**Age at diagnosis**	68 (59–74)
**Smoking status**^b^	
Current	61 (39%)
Former	74 (47%)
Never	20 (13%)
Unknown	1
**Follow-up time (yr)**^c^	6.8 (3.5–9.5)
**HG recurrence within 2-yr follow-up (HG-rec)**	
FU <2 yr^d^	9 (5.8%)
Yes	62 (40%)
**EAU risk group**	
Intermediate risk	57 (37%)
High risk	64 (41%)
Very high risk	35 (22%)
**Days from PDD to BCG**^e^	51 (30–176)
**PDD status**^f^	
No PDD	85 (54%)
Over 100 d	22 (14%)
Within 100 d	49 (31%)

BCG = Bacillus Calmette-Guérin; EAU = European Association of Urology; FU = follow-up; HG = high grade; PDD = photodynamic diagnosis; RNA = ribonucleic acid.

a n (%); Median (Q1–Q3).

b At the time of inclusion in study.

c From pre-BCG RNA-sequenced tumor to end of follow-up.

d Died within 2 yr of FU. Patients without an event and less than two years of FU after BCG were excluded from binary outcome analyses.

e Of patients who have received PDD at any time before BCG.

f Days from PDD to BCG initiation.

### 3.2 Urine proteomics characterization of PDD-groups

Using a 92-protein Olink proteomics panel, we investigated the proteomic profile of 60 paired urine samples (19 PDD and 41 No PDD) to assess if there was a difference in relation to PDD exposure. The mean time between pre- and post-BCG samples was 4.5 mo (range: 3.0–6.9 mo). The difference in normalized protein expression unit (NPX) values between pre- and post-BCG samples were mapped and stratified according to PDD exposure (Fig. 1B).

To focus on proteins with higher relative expression levels, NPX values were filtered above the protein-specific median prior to analysis. Subsequent evaluation of individual protein expression revealed significant pre- to post-BCG treatment changes in a subset of proteins within either the PDD group or No PDD group. These proteins were selected for visualization to illustrate differential treatment responses (Fig. 1C). In the PDD group we found a significant decrease in CASP-8 and CXCL5 and an increase in CXCL10, MCP-4, PDCD1 (PD-1), and VEGFR-2 after BCG treatment. In the No PDD group we observed a significant increase in CD5, HGF, and LAMP3 after BCG treatment.

**Fig. 2:**
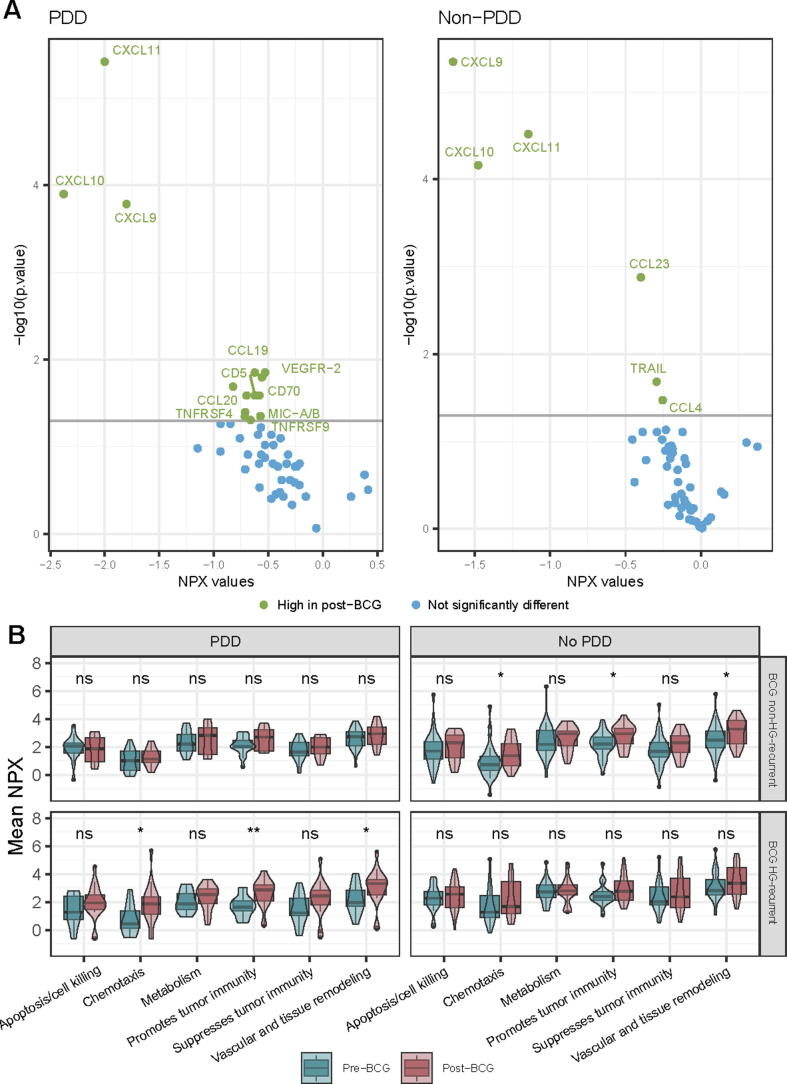
Urinary profile. (A) Volcano plots of paired pre- and post-BCG samples. All significantly different proteins are named (Paired Wilcoxon signed-rank test). Green = significant Benjamini Hochberg–adjusted p value (q value) <0.1. Blue = not significant. Gray lines indicate a significance level of 0.05. PDD-group (*n* = 25) and No-PDD group (*n* = 41). (B) NPX grouped by biological features. Comparison of mean NPX grouped by biological features between pre- and post-BCG samples for PDD and No-PDD samples (Wilcoxon rank-sum test) with unadjusted p values stratified by post-BCG recurrence status. PDD+No HG-rec (*n* = 26), PDD+HG-rec (*n* = 32), No-PDD+No HG-rec (*n* = 56), and No-PDD+HG-rec (*n* = 55). Significant differences are denoted with *, ** or *** if they are <0.05, <0.01 or <0.001, respectively. BCG = Bacillus Calmette-Guérin; NPX = normalized protein expression unit; PDD = photodynamic diagnosis; ns = not significant.

Based on this, we wanted to compare differentially expressed proteins in the paired samples to estimate which proteins were upregulated after BCG. In both groups there was an upregulation of CXCL9, CXCL10, and CXCL11 after BCG treatment, indicating a general immune activation. Patients in the PDD group had higher post-BCG levels of receptors of the tumor necrosis factor (TNF)-superfamily (TNFRSF), such as TNFRSF12A, TNFRSF4, and TNFRSF9, which play a role in T-cell activation [17]. Immunoinhibitory proteins such as CD70, PD-1 (PDCD1), and CD5 were also observed in the PDD group after BCG. In the No PDD group CCL23, TRAIL and CCL4 were significantly higher post-BCG (Fig. 2A).

When proteins were grouped according to biological function, comparative analysis revealed that BCG treatment induced changes in specific biological processes, including chemotaxis, tumor-promoting immune responses, and vascular tissue remodeling. Notably, these changes were observed in patients with HG recurrence within the PDD group, and in patients without HG recurrence within the No PDD group (Fig. 2B). In contrast, the comparison of post-BCG samples between the PDD and No PDD groups did not reveal statistically significant differences (Supplementary Fig. 4).

### 3.3 Characterization of tumor microenvironment changes between PDD-groups

**Fig. 3:**
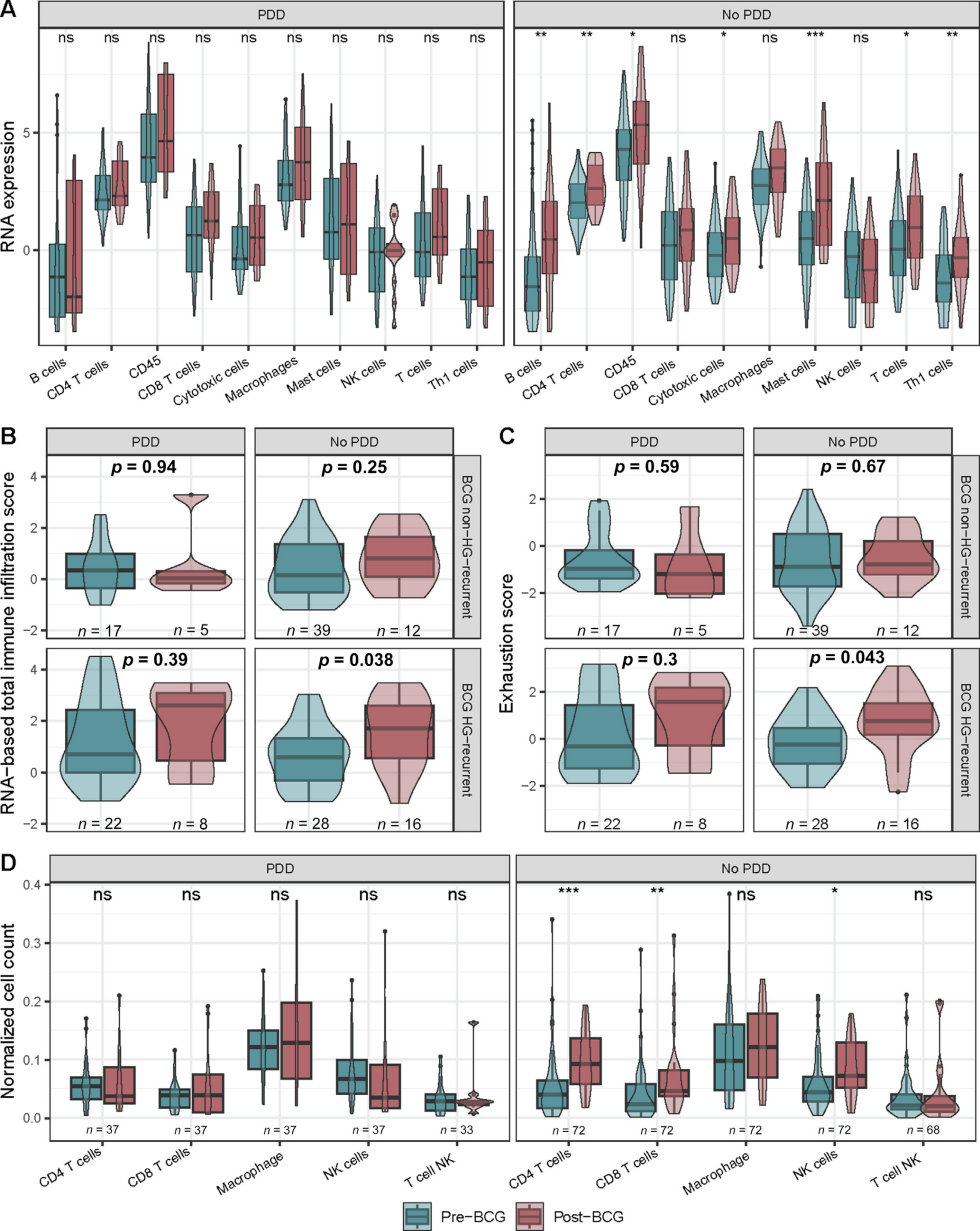
Characterization of tumor microenvironment through transcriptomic and IMC data. (A) Comparison of RNA-based immune subsets before and after BCG stratified by PDD status. PDD (*n* = 53) and No-PDD (*n* = 96). (Wilcoxon rank-sum test, with unadjusted p values) (B) RNA-based immune cell infiltration in pre- and post-BCG samples from the groups with and without post-BCG HG recurrence (Wilcoxon rank-sum test) (C) Post-BCG T cell exhaustion. Comparison of level of T cell Exhaustion between pre- and post-BCG samples stratified by PDD status and BCG response level (Wilcoxon rank-sum test). (D) IMC data. Comparison of selected immune subsets from IMC between pre- and post-BCG samples stratified by PDD and BCG response status (Wilcoxon rank-sum test). Significant differences are denoted with *, **, or *** if they are <0.05, <0.01 or <0.001, respectively. BCG = Bacillus Calmette-Guérin; NPX = normalized protein expression unit; PDD = photodynamic diagnosis; ns = not significant.

Next, we investigated if the local tumor microenvironment was affected by PDD. A total of 108 pre-BCG and 41 post-BCG samples were included in the RNA-seq analyses. We used the RNA-seq data to assess if RNA-based immune subsets, based on established gene expression signatures [18], were differentially expressed based on PDD status after BCG. In the No PDD group, comparison of pre- and post-BCG samples revealed a significant increase in B cells, CD4⁺ T cells, CD45⁺ cells, cytotoxic cells, mast cells, total T cells, and Th1 cells following BCG treatment (Fig. 3A). No significant changes were observed in the PDD group. When stratifying by post-BCG recurrence status, the results were slightly altered: in the No PDD group with HG recurrence, CD4⁺ T cells, mast cells, neutrophils, and regulatory T cells (Tregs) increased post-BCG. In contrast, Th1 cells increased only in the No PDD group with no HG recurrence (Supplementary Fig. 5).

The immune infiltration score, calculated as the mean of all included immune cell types, increased after BCG treatment in the No PDD group among patients with HG recurrence (*p* = 0.038), an effect that was not observed in the other subgroups (Fig. 3B). Analysis of a previously established CD8⁺ T-cell exhaustion score revealed higher exhaustion in patients who did not receive PDD within the BCG HG recurrent group (*p* = 0.043), whereas these changes were not observed in the other groups (Fig. 3C).

Using IMC data from 125 tumors, we characterized the protein expression profiles of distinct immune cell populations within the tumor microenvironment before and after BCG treatment. In the No PDD group, we observed a post-treatment increase in the abundance and of CD4⁺ T cells, CD8⁺ cytotoxic T cells, and natural killer cells, suggesting an enhanced antitumor immune response following BCG instillation. In contrast, the PDD group showed no significant alterations in the expression of these immune cell subsets (Fig. 3D).

## 4 Discussion

Our study revealed differences in the urinary protein profile between patients who received PDD and those who did not. Specifically, chemokines such as CXCL10 and MCP-4, along with immune-related markers PDCD1 (PD-1) and VEGFR-2, were upregulated in urine after BCG in PDD-exposed patients. CXCL10 and MCP-4 are potent chemotactic cytokines involved in T-cell and monocyte recruitment [19], [20], while VEGFR-2 reflects vascular remodeling and endothelial activation [21], consistent with an acute inflammatory or reparative response. The concurrent increase in PDCD1 suggests activation-induced checkpoint expression, potentially reflecting transient immune engagement rather than sustained exhaustion [22]. Analysis of differentially expressed proteins showed that CXCL9, CXCL10, and CXCL11 were upregulated after BCG in both groups, consistent with known BCG-induced immune activation [15], [19]. In the PDD group, additional increases in TNFRSF12A, TNFRSF4, and TNFRSF9 suggest activation of costimulatory pathways and local inflammatory signaling, consistent with a surface-confined luminal immune response [17]. These findings may represent a luminal immune reaction confined to the bladder surface, where photodynamic exposure occurs, rather than an intratumoral change.

Analysis of the TME through RNA-seq and IMC revealed an increase in immune cell infiltration, particularly CD4+ and CD8+ T cells, in the No PDD group after BCG treatment. This may reflect a more pronounced local immune response in these patients, which was not seen in the PDD group. However, it remains uncertain whether such differences are directly related to PDD exposure, as the tumor samples used for RNA analysis, both pre- and post-treatment, were collected much further from the time of BCG and PDD exposure than the urine samples. Consequently, temporal differences in sampling may partly explain the discrepancies observed between tissue and urine analyses. Given the non-randomized design and modest magnitude of the observed changes, these results should be interpreted cautiously, as some differences may also have occurred by chance.

Sample timing varied considerably across data types. Urine was collected near PDD and BCG exposure, capturing short-term immune responses, whereas RNA-seq tumor samples were obtained months before or after treatment. Tumor samples used for TMAs (IMC analyses) were closer to BCG but still less temporally aligned than urine. Consequently, urinary changes likely reflect transient immune activation, while tumor data represent longer-term states less likely directly linked to PDD.

Patients who received PDD prior to BCG were likely selected based on higher perceived risk, such as larger or multiple tumors or prior recurrences, introducing potential selection bias. This makes it difficult to separate effects of PDD from underlying risk factors. After adjustment for EAU risk group, age, and sex, no statistically significant association was observed between PDD status and outcome. The mix of paired and unpaired samples adds heterogeneity, which may affect detection of treatment-related changes. To reduce temporal variability, we excluded samples collected more than 100 days after PDD, though the optimal window for PDD’s biological effect remains unknown.

Few studies have examined the direct effects of PDD on the bladder TME [12], [13]. Most research has focused on PDT, which uses higher light doses and longer wavelengths to induce ROS-mediated cell death [23], [24]. While both PDD and PDT rely on light activation of photosensitizers like HAL, PDD uses lower-intensity light optimized for fluorescence, not cytotoxicity, so no ROS-mediated tissue damage occurs. As a result, PDD studies primarily address diagnostic improvements rather than direct biological effects on the TME. To our knowledge, Elbæk et al. [13] conducted the only study that investigated immune-related changes in the TME between PDD (HAL-BLC) and white-light cystoscopy in human bladder cancer. They observed an increase in cytotoxic T lymphocytes (CTLs) over time in patients who underwent PDD, an effect not seen in the white-light group. Moreover, when comparing changes from TURBT to radical cystectomy, PDD was associated with a decrease in B cells, CTLs, and PD-1 expression in the stromal compartment, suggesting a distinct modulatory impact on the immune microenvironment compared to white light alone. Unfortunately, we did not observe similar trends in our cohort, where the main proteomic changes of the tumors were observed in the No PDD group.

Limitations of our study include a retrospective study design and relatively small sample size within subgroups—particularly those experiencing HG recurrence. Sample availability constrained the number of post-BCG tumors analyzed by RNA-seq and IMC, which may have limited sensitivity for detecting subtle immune changes. Furthermore, Olink proteomics provides relative rather than absolute protein quantification and reflects soluble proteins shed into urine; these may not directly mirror intratumoral processes.

## 5 Conclusion

This exploratory study suggests that prior PDD exposure is associated with distinct immune-related urinary proteomic changes after BCG, while intratumoral immune remodeling appears more pronounced in patients not exposed to PDD. Whether these differences reflect biological effects of PDD or residual clinical confounding remains unclear. Given the observational design and potential confounding, these findings do not allow conclusions regarding direct immunological effect of PDD, especially considering no consistent tumor-level immune signal in association to PDD was detected. Overall, our findings support that the established clinical benefit of PDD may be more related to improved tumor detection rather than a direct modulation of the tumor immune microenvironment.

  ***Author contributions:*** Lars Dyrskjøt had full access to all the data in the study and takes responsibility for the integrity of the data and the accuracy of the data analysis.

  *Study concept and design*: Andreasen, Young-Halvorsen, Neijber, Dyrskjøt.

*Acquisition of data*: Andreasen, Strandgaard, Divita.

*Analysis and interpretation of data*: Andreasen, Strandgaard, Divita, Young-Halvorsen, Neijber, Dyrskjøt.

*Drafting of the manuscript*: Andreasen, Strandgaard, Dyrskjøt.

*Critical revision of the manuscript for important intellectual content*: Andreasen, Strandgaard, Divita, Young-Halvorsen, Neijber, Dyrskjøt.

*Statistical analysis*: Andreasen.

*Obtaining funding*: Dyrskjøt.

*Administrative, technical, or material support*: Dyrskjøt.

*Supervision*: Dyrskjøt.

*Other* (specify): None.

  ***Financial disclosures:*** Lars Dyrskjøt certifies that all conflicts of interest, including specific financial interests and relationships and affiliations relevant to the subject matter or materials discussed in the manuscript (eg, employment/affiliation, grants or funding, consultancies, honoraria, stock ownership or options, expert testimony, royalties, or patents filed, received, or pending), are the following: Anders Neijber: Employee of Photocure and hold stock or stock options in Photocure ASA.

Kristine Young-Halvorsen: Employee of Photocure and hold stock or stock options in Photocure ASA.

Lars Dyrskjøt: Sponsored research agreements with C2i Genomics, Natera, AstraZeneca, Photocure, and Ferring and serves in an advisory/consulting role for Ferring, MSD, Cystotech, and UroGen. LD has received speaker honoraria from AstraZeneca, Pfizer, and Roche, as well as travel support from MSD.

No disclosures were reported by the other authors.

  ***Funding/Support and role of the sponsor:*** None.

  ***Ethics statement:*** This study was approved by the Danish National Ethics Committee (#1708266).

  ***Data sharing statement:*** The current legislation on data sharing requires that sensitive (including pseudonymized) data can only be shared following approval by the National Committee on Health Research Ethics in Denmark of the project and by the Danish Data Registry.
