# Supplementary material for: Impact of Photodynamic Diagnosis on Immune Cell Infiltration in BCG-treated Non–muscle-invasive Bladder Cancer
Source: Eur Urol Open Sci. 2026 Aug 8;91:74–83. doi: 10.1016/j.euros.2026.07.006 (PMC13476646; doi:10.1016/j.euros.2026.07.006)
Supplement: Supplementary file 1 [file mmc1.docx]

## Supplementary data

**Supplementary figure 1:** Detailed overview of patients disease courses and analyzed samples. Lines represent patient’s disease courses from time of first analyzed tumor or urine sample to end of follow-up centered around the first induction treatment of BCG with at least five instillations. Colors indicate performed analyses. Shapes indicate the number of analyses per clinical timepoint. Left: full follow-up. Right: Two years pre- and post-BCG illustrated.

**Supplementary figure 2:** Histogram showing the number of days from PDD to BCG treatment for all patients who at any point had received PDD before BCG. The vertical line represents 100-day cut-off.

**Supplementary Figure 3:** Forest plot of multivariable Cox regression analysis for recurrence-free survival. Dots represent hazard ratios and horizontal lines the corresponding 95% confidence interval (CI). Age is modeled as a continuous variable with the hazard ratio reported per 1-year increase. *PDD = Photodynamic Diagnosis; EAU = European Association of Urology.*

**Supplementary figure 4:** Post-BCG samples. Comparison of mean NPX value between PDD groups (Wilcoxon rank-sum test, with unadjusted p values).

**Supplementary figure 5:** RNA-based immune subsets before and after BCG stratified by PDD status and post-BCG response (Wilcoxon rank-sum test, with unadjusted p values). Significant differences are denoted with *, ** or *** if they are < 0.05, < 0.01 or < 0.001, respectively.

| **Characteristic** | **PDD negative**  N = 85*^a^* | **PDD positive**  N = 49*^a^* | **p-value***^b^* |
| --- | --- | --- | --- |
| **Sex (male)** | 68 (80%) | 38 (78%) | 0.7 |
| **Age at diagnosis** | 65 (59, 73) | 68 (60, 73) | 0.4 |
| **Smoking Status*^c^*** |  |  | 0.090 |
| Current | 40 (47%) | 14 (29%) |  |
| Former | 36 (42%) | 28 (57%) |  |
| Never | 9 (11%) | 6 (12%) |  |
| Unknown | 0 | 1 |  |
| **Follow-up time (years)*^d^*** | 7.4 (3.9, 10.3) | 6.6 (3.4, 8.4) | 0.13 |
| **HG recurrence within 2-year follow-up (HG-rec)** |  |  | 0.5 |
| FU<2yr | 5 (5.9%) | 3 (6.3%) |  |
| No | 48 (56%) | 22 (46%) |  |
| Yes | 32 (38%) | 23 (48%) |  |
| **EAU risk group** |  |  | **<0.001** |
| Intermediate Risk | 41 (48%) | 8 (16%) |  |
| High Risk | 26 (31%) | 26 (53%) |  |
| Very High Risk | 18 (21%) | 15 (31%) |  |
| **Days from PDD to BCG** | NA | 38 (28, 53) |  |
| *^a^*n (%); Median (Q1, Q3) | | | |
| *^b^*Pearson's Chi-squared test; Wilcoxon rank sum test; Fisher's exact test | | | |
| *^c^*At the time of inclusion in study | | | |
| *^d^*From pre-BCG RNA-sequenced tumor to end of follow-up | | | |

**Supplementary table 1:** Baseline characteristics stratified by PDD status. PDD status status is based on a 100 day cut-off between PDD and BCG initiation. *BCG = Bacillus Calmette-Guérin; PDD = Photodynamic Diagnosis, HG-rec = High Grade recurrence; EAU = European Association of Urology.*

| ***Characteristic*** | ***HR*** | ***95% CI*** | ***p-value*** | ***p-adjust^a^*** |
| --- | --- | --- | --- | --- |
| *PDD status (No-PDD vs PDD)* | *0.75* | *0.49, 1.15* | *0.2* | *0.2* |
| *EAU risk group (High/Very High vs Intermediate)* | *1.36* | *0.88, 2.11* | *0.2* | *0.2* |
| *Age at diagnosis* | *1.00* | *0.99, 1.02* | *0.6* | *0.6* |
| *Sex (female vs male)* | *0.63* | *0.40, 1.02* | *0.069* | *0.2* |
| *^a^False discovery rate correction for multiple testing* | | | | |

**Supplementary table 2:** Cox proportional hazards regression analysis for recurrence-free survival. Hazard ratios are calculated from multivariable Cox regressions. Age is modeled as a continuous variable with HR reported per 1-year increase. *PDD = Photodynamic Diagnosis; EAU = European Association of Urology; CI = Confidence Interval; HR = Hazard Ratio.*
